# F188. THALAMIC MICROSTRUCTURE IN UNAFFECTED RELATIVES OF SCHIZOPHRENIA

**DOI:** 10.1093/schbul/sby017.719

**Published:** 2018-04-01

**Authors:** Wu Jeong Hwang, Kang Ik Cho, Yoo Bin Kwak, Tae Young Lee, Jun Soo Kwon

**Affiliations:** 1 Seoul National University; 2 Seoul National University, Medical Research Center; 3 Seoul National University College of Medicine

## Abstract

**Background:**

Family, twin, adoption and candidate gene studies all support a genetic component for psychotic disorders. A considerable evidence suggests that the thalamus is abnormal in schizophrenia. The thalamus has a heterogeneous structure with its nucleus having distinct inputs and outputs. Disrupted thalamo-cortical connectivity, in particular, is considered as a core psychopathology in patients with schizophrenia. The disruption is also observed in subjects at clinical-high risk for psychosis. However, using the conventional magnetic resonance imaging methods, it had been difficult to investigate the subtle structural changes that may be present in the thalamus. Furthermore, despite the numerous reports of thalamic abnormalities in schizophrenia, the genetic aspect of the thalamic microstructure has not been thoroughly investigated.

**Methods:**

To examine the microstructure of the thalamus, a total of 34 unaffected relatives of schizophrenia (UR) and 33 healthy control subjects underwent diffusion-weighted and diffusion kurtosis magnetic resonance imaging. Using the probabilistic tractography the projections from the thalamus to the lateral and medial prefrontal cortices, lateral and medial temporal cortices, occipital cortex, somatosensory cortex, parietal cortex and orbitofrontal cortex were analyzed. Then, the thalamus was segmented by the projections and the microstructures of those segmented regions were compared between the groups. The mean kurtosis values of the segmented regions were analyzed by analysis of covariance with age and sex as covariates and the results were adjusted with Bonferroni correction.

**Results:**

There was no statistical difference in the mean kurtosis values of the left and right thalamic regions projecting to any of the investigated regions between the UR and healthy controls.

**Discussion:**

Our findings, via diffusion kurtosis imaging, show preserved microstructural integrity of the thalamus in UR and that this imaging technique may be less well suited to detect thalamic abnormalities in them.

